# Third-Trimester Cerebroplacental Ratio and Preceding Fetal Growth in Appropriate-for-Gestational-Age Fetuses: Limited Individual-Level Information in a Longitudinal Cohort

**DOI:** 10.3390/diagnostics16213442

**Published:** 2026-10-22

**Authors:** Maria Cezara Mureșan, Marius Bogdan Mureșan, Dan Dumitrascu-Biris, Zoran Laurențiu Popa, Cosmin Citu

**Affiliations:** 1 Department of Obstetrics and Gynecology, Faculty of Medicine, Victor Babeș University of Medicine and Pharmacy, 300041 Timișoara, Romaniacitu.ioan@umft.ro (C.C.); 2 Maternal-Fetal Medicine Center, 300331 Timișoara, Romania

**Keywords:** cerebroplacental ratio, fetal growth velocity, conditional growth, Doppler ultrasound, appropriate for gestational age, measurement error

## Abstract

**Background/Objectives:** Cerebroplacental ratio (CPR) is mainly a contemporaneous hemodynamic and prognostic marker but has also been proposed as a marker of impaired growth velocity. We quantified how much third-trimester CPR constrains an individual fetus’s preceding measured growth. **Methods:** In a retrospective single-center cohort, conditional estimated fetal weight growth (cEFW) was derived in 920 singleton pregnancies and analyzed in 760 fetuses appropriate for gestational age (AGA) at the third-trimester scan (median 32.9 weeks), with umbilical and middle cerebral artery Doppler measurements obtained during the same examination. Quantile regression estimated the conditional/unconditional 10th–90th percentile width of cEFW (width ratio) by within-cohort CPR z-score. **Results:** Each 1 SD increase in CPR corresponded to 0.100 SD higher cEFW (95% CI 0.040–0.160); the AUC for cEFW ≤ −1 SD was 0.553 (0.479–0.619). Point-estimate width ratios ranged from 0.968 to 1.194 between CPR z −2 and +1, with wide intervals at low CPR. The modeled probability of cEFW ≤ −1 SD was 14.4% (8.5–22.1) at z −2 and 10.5% (8.4–12.9) at z 0. Without AGA restriction (*n* = 878), the association was stronger (AUC 0.615), but point-estimate width ratios likewise showed no narrowing at low CPR. **Conclusions:** In AGA fetuses scanned mainly at 31–34 weeks, CPR provided limited individual-level information about preceding measured growth in terms of central 80% width, discrimination and lower-tail probabilities; at very low CPR, meaningful narrowing cannot be excluded. Prognostic value for perinatal outcome was not assessed.

## 1. Introduction

Late fetal growth restriction cannot be defined by size alone. Consensus definitions combine fetal size, crossing of growth centiles and Doppler evidence of altered fetoplacental hemodynamics, treating these as distinct contributory features rather than interchangeable measurements [1,2,3,4]. Serial biometry describes change over time, whereas Doppler describes the hemodynamic state at the examination during which it is obtained.

The cerebroplacental ratio (CPR), calculated from middle-cerebral-artery and umbilical-artery pulsatility indices, is used and studied primarily as a contemporaneous marker of fetal hemodynamic adaptation and as a prognostic marker for adverse perinatal outcome [5,6,7,8,9,10]. A separate literature has reported lower average CPR in fetuses with reduced growth velocity, including fetuses that remain within the appropriate-for-gestational-age (AGA) range, and has described CPR as a possible marker of impaired growth velocity or failure to reach growth potential [11,12,13,14]. That wording does not mean that clinical guidelines recommend CPR as a substitute for serial biometry; rather, it raises a distinct information question about how strongly a single CPR value constrains the measured growth trajectory that preceded it.

This distinction matters because population association and individual information are not equivalent [15]. If fetuses that decelerate have a lower CPR on average, CPR may still have substantial overlap between stable and decelerating trajectories. Conversely, a marker can carry useful prognostic information about perinatal outcome without reconstructing a previous biometric trajectory. We therefore do not test whether CPR predicts placental insufficiency, intrapartum compromise or neonatal morbidity; those are different clinical questions.

Previous longitudinal work provides an important context. In the Pregnancy Outcome Prediction (POP) study, universal third-trimester biometry identified more small-for-gestational-age infants than selective scanning, and low abdominal-circumference growth velocity helped identify a subgroup of small fetuses at increased neonatal risk [16]. Studies in normally sized fetuses likewise linked reduced growth velocity with placental insufficiency or adverse characteristics [11,12,13,14]. In parallel, systematic reviews and meta-analyses have shown heterogeneous prognostic performance of CPR for adverse perinatal outcomes [5,6,7,9,10]. These studies establish biological and prognostic associations, but they do not directly quantify how much a given CPR value narrows the range of preceding measured growth histories in an individual fetus.

Two methodological issues are particularly important for that question. First, change between two ultrasound examinations is affected by baseline size and regression to the mean, which conditional growth methods are designed to address [17,18,19]. Second, both serial biometry and CPR are measured with error, and CPR also varies with gestational age and with the reference chart used [9,20,21,22,23,24,25,26,27]. We therefore asked how much a gestational-age-standardized third-trimester CPR narrows the central distribution of preceding measured growth histories in AGA fetuses, conditional on baseline size, while explicitly examining model specification, gestational timing, external CPR scaling and measurement error.

## 2. Materials and Methods

### 2.1. Study Design, Dating and Ultrasound Protocol

This retrospective longitudinal cohort study used prospectively recorded examinations stored in the ASTRAIA database of a maternal–fetal medicine center in Timișoara, Romania. The study database was closed for this analysis on 6 August 2026, the date of the latest examination included in the analytic dataset. The parent dataset comprised 8985 selected singleton pregnancies with first-trimester screening performed in the center. Gestational age was established from first-trimester crown–rump length measured at the center; last menstrual period was not used for dating and was not entered into the research dataset. Examinations were performed by three senior maternal–fetal medicine specialists, all certified by the Fetal Medicine Foundation; two also held an FMF Diploma, and all three held Romanian subspecialty certification in maternal–fetal medicine. The ultrasound systems used during the study period were Voluson 730, E6, E10 and Expert 22 (GE Healthcare).

A third-trimester growth scan at approximately 32–34 weeks was routinely offered at the center. At that visit, clinicians reviewed relevant findings and risk markers documented at the first- and second-trimester examinations, but the recommendation for the T3 examination itself was routine rather than contingent on suspected fetal growth restriction or another previously identified abnormality. Third-trimester examinations included systematic Doppler assessment of the umbilical artery (UA), middle cerebral artery (MCA) and ductus venosus (DV). DV was not used in the present analyses. The database export stores the final clinically recorded biometric and Doppler values, but not waveform-level replicates, the number of repeated caliper measurements, or the identity of the T2/T3 operator. Therefore, whether the same operator performed both examinations and the number of repeated traces from which a final value was chosen cannot be reconstructed from the archived export. Previous examinations were available and routinely reviewed as part of clinical care; however, the archived export does not document which specific prior findings were actively considered during each T3 examination. This limitation is relevant to the interpretation of measurement error.

Second-trimester (T2) biometry was selected within 20 + 0–24 + 6 weeks as the examination closest to 22 + 0 weeks; third-trimester (T3) biometry was selected within 28 + 0–41 + 6 weeks as the examination closest to 34 + 0 weeks. The selected visit was retained before applying the reference-domain restriction; no later alternative visit was substituted. Of 8985 parent pregnancies, 6598 had eligible T2 biometry and 2551 also had eligible T3 biometry. Eleven records were then removed by the frozen cleaning rules, leaving 2540 longitudinal records. Restriction to T2 ≥ 22 + 0 weeks, required by the EFW reference domain, left 920 pregnancies; none was additionally excluded for T3 > 40 + 0. Of these, 800 were AGA at T3 (EFW 10th–90th centile), and 760 from 745 women had positive same-examination UA-PI and MCA-PI (Table 1; Figure S1). The 8985 parent pregnancies were themselves screening-selected rather than all pregnancies attending the center.

### 2.2. Cohort Selection, Analysis Plan and Related Work

The operational analysis plan was frozen on 28 September 2026 before the primary quantile-regression and bootstrap analyses. The question arose from an earlier internal exploratory analysis of the same database; that exploratory work was not a separate publication or submission. Because the 10th–15th band of the internally standardized CPR distribution had already been inspected, it is retained only for descriptive illustration and is not used for inference. The plan documented the valid EFW reference domain, the conditional-variance decision rule and the use of internal CPR standardization when the intended external calibration could not be implemented reproducibly from the archived source materials. The measurement-error simulation and the uterine-artery/maternal-characteristic models were developed after the primary result and are labeled accordingly. Two other manuscripts from the same clinical database address prenatal screening and genetic diagnosis in differently assembled samples; neither addresses the present CPR–growth question, and no tables or figures are shared.

### 2.3. Growth Variables

EFW was calculated with the INTERGROWTH-21st formula based on abdominal circumference (AC) and head circumference (HC) [28] and converted to INTERGROWTH-21st z-scores. In the 920-pregnancy derivation cohort, let Z2 and Z3 denote the T2 and T3 EFW z-scores and let t = (GA3 − GA2)/7 − 11, where gestational ages are in days and t is the interval in weeks centered at 11 weeks. The fitted mean model was: E[Z3|Z2,t] = −0.0126330456022 + 0.441554538936 × Z2 − 0.0409341998279 × t − 0.0260343436815 × Z2 × t. The interaction allows the association with baseline size to vary with the time between scans; it is a statistical adjustment rather than a biological model. The mean model was estimated before restriction to AGA fetuses, avoiding derivation of expected T3 size in a sample already truncated on T3 size [17,18].

The residual-variance model was a gamma generalized linear model with log link fitted to squared residuals: V(t) = exp(−0.555588134885 − 0.0650160068301 × t). Conditional growth was cEFW = [Z3 − E(Z3|Z2,t)]/sqrt[V(t)]. The frozen plan specified interval-specific scaling when the ratio of the 90th to the 10th percentile of predicted residual SD was ≥1.10; the observed ratio was 1.10757. The variance-slope *p* value was 0.0848 and Levene’s test *p* = 0.292, so the rationale for conditional scaling is the prespecified rule rather than evidence of strong heteroscedasticity. In the derivation cohort, cEFW had a mean of −0.00004, SD 1.0005, skewness of −0.069 and excess kurtosis of 0.900; normality was not assumed by the quantile-regression analysis. A joint linear/quadratic test found no residual dependence of mean cEFW on T3 gestational age before AGA restriction (*p* = 0.909); after AGA restriction, a small association remained (*p* = 0.0258). A sensitivity analysis therefore added linear and quadratic T3 gestational-age terms to the mean model.

As a complementary published method, AC velocity was calculated as the INTERGROWTH-21st conditional SDS, (Z_2_ − r_12_Z_1_)/√(1 − r_12_^2^), with r_12_ from the published longitudinal correlation model [18,19]. Because AC contributes to EFW, this is a methodological check rather than biologically independent validation.

### 2.4. Cerebroplacental Ratio

CPR was calculated as MCA-PI/UA-PI [29] from the same selected T3 examination. The internal reference model used one selected T3 examination per pregnancy from 2592 pregnancies representing 2408 women; 181 women contributed more than one pregnancy, but no pregnancy contributed repeated examinations to the reference model. Among these 2592 pregnancies, 141 (5.44%) had T3 EFW < p10 and 181 (6.98%) had T3 EFW > p90. Chronic hypertension was recorded in 37/2592 (1.43%), but diabetes and pre-eclampsia fields were not sufficiently complete or consistently coded to estimate a reliable overall proportion of complicated pregnancies. Although 346 reference pregnancies had more than one raw CPR-containing visit in the source export, only the prespecified selected visit was used for calibration.

For internal standardization, let g = GA3/7 − 34. The fitted mean of log(CPR) was m(g) = 0.726072044370 − 0.00630950082760 × g − 0.00463599010505 × g^2^, with residual SD 0.257755798253; CPR z = [ln(CPR) − m(g)]/0.257755798253. This is a within-cohort study standardization and must not be interpreted as a validated clinical CPR centile. The originally intended external calibration could not be implemented reproducibly from the archived materials because the numerical transformation required for the prespecified chart was not available in a form that could be independently audited; no coefficients were inferred from plots or approximate values. During revision, we therefore added an external sensitivity analysis based on the fully published week-specific CPR medians of Zohav et al. [25], expressing CPR as MoM using both completed gestational week and linear interpolation between published weekly medians. The sensitivity analysis is intended as an external scaling check, not as validation of a universal clinical threshold, because published CPR charts differ materially [9,24,25,26,27].

### 2.5. Statistical Analysis

Primary analysis. Quantile regression (τ = 0.10, 0.50, 0.90) of cEFW on CPR z was modeled by a restricted cubic spline with knots at the 5th, 35th, 65th and 95th percentiles of CPR z and fitted by linear programming. At CPR z −2, −1.5, −1, 0 and +1, the conditional P10–P90 width was divided by the unconditional P10–P90 width in the same AGA cohort. This width ratio quantifies the dispersion of the central 80% of measured growth histories; it does not summarize the conditional median, tail-specific changes or all information contained in the distribution. These features were therefore reported separately. A ratio of 1 denotes no change in central 80% width at the evaluated CPR value. No equivalence margin was prespecified, and confidence intervals were not interpreted as equivalence tests. Pointwise 95% confidence intervals were percentile intervals from 2000 woman-level cluster bootstrap replicates. The resampling frame comprised all 2717 pregnancies of 2519 women that belonged to at least one analysis set (the 2592-pregnancy CPR reference, the 920-pregnancy cEFW derivation cohort or the frozen longitudinal set); women were sampled with replacement, and all pregnancies of a sampled woman were carried with their fixed eligibility flags. Within each replicate, CPR z was re-standardized on the resampled reference pregnancies, the cEFW mean and variance models were refitted on the resampled derivation pregnancies, and the primary AGA set, spline knots and unconditional denominator were recomputed, so that the overlapping sets were always drawn from the same resampled women. The same analysis was repeated for conditional AC velocity.

Secondary analyses. Mean association was estimated by linear regression with standard errors clustered on the woman. Individual discrimination was summarized by the AUC of −CPR z for cEFW ≤ −1 SD and ≤ −1.5 SD. AUC confidence intervals were percentile intervals from the same 2000 bootstrap replicates. Multiple pregnancies of the same woman (15 women with two pregnancies in the primary set) were retained and handled by woman-level clustering in both the regression standard errors and the bootstrap; a one-pregnancy-per-woman sensitivity analysis was also performed. These two thresholds were chosen as descriptive standardized lower-tail cutoffs of increasing severity, not as validated definitions of pathological growth slowing; they do not diagnose fetal growth restriction, placental insufficiency or adverse perinatal outcome. The more extreme threshold was expected to yield fewer events and was interpreted accordingly. During revision, we added the change in the conditional median between CPR z −2 and +1 and modeled probabilities of cEFW ≤ −1 SD and ≤−1.5 SD at the five evaluated CPR values, estimated by logistic regression with a linear CPR z term; their confidence intervals came from the same bootstrap replicates and seeds as the primary analysis.

Sensitivity analyses included all fetal-size classes, one pregnancy per woman, calendar epochs, a narrower T2–T3 interval, global rather than interval-specific residual SD, the wider historical examination window, and a mean model additionally adjusted for linear and quadratic T3 gestational age. Spline sensitivity used 3 knots (p10, p50, p90), the primary 4 knots (p5, p35, p65, p95), alternative 4-knot tail placements (p2.5/p97.5 and p10/p90) and 5 knots (p5, p27.5, p50, p72.5, p95). To address gestational timing, the primary analysis was repeated in T3 examinations at ≥34 weeks; the ≥36-week subset was also enumerated before attempting inferential modeling. The external CPR sensitivity used Zohav CPR MoM as described above. Because AGA status was defined from T3 EFW, which also enters cEFW, the analysis of all fetal-size classes was additionally bootstrapped within the same replicates and is reported in the main text, together with the raw CPR distribution and the numbers of pregnancies below the external 5th and 10th percentiles.

Measurement-error simulation. Serial EFW was simulated for fetuses that exactly tracked their latent z-score between examinations, with added ultrasound error, and then passed through the same conditional construction and AGA selection. Nineteen scenarios, each with 500 simulations, varied random EFW error, error derived from published intra- and interobserver variability of AC and HC, the between-examination correlation of errors and latent dispersion [21,22]. For each scenario, we compared simulated error-only cEFW variance with observed cEFW variance and approximated, under an additional Gaussian signal/noise model, the width ratio attainable by an ideal predictor of error-free growth. These quantities are assumption-dependent benchmarks, not identified error fractions or clinical bounds. The simulation addressed biometric error only and did not model CPR measurement error.

Analyses used Python 3.12 with NumPy 2.5.3, pandas 3.0.6, SciPy 1.18.1 (HiGHS solver) and statsmodels 0.15.0. The primary-analysis, measurement-error and revision-analysis scripts and aggregate outputs are provided in Supplementary Code; no individual-level clinical records are included, in accordance with the Data Availability Statement.

Use of generative artificial intelligence. ChatGPT and Codex (OpenAI) and Claude (Anthropic) were used to assist with data-processing and analysis scripts, computational cross-checks, and drafting and editing. The tools were used during manuscript preparation and revision in September–October 2026. Exact model-version identifiers were not retained. The authors specified the analyses, reviewed the code and outputs, and take responsibility for the reported numbers and final text. 

### 2.6. Ethics

This study was conducted in accordance with the Declaration of Helsinki and approved by the Ethics Committee of the Maternal-Fetal Medicine Center, Timișoara, Romania. The initial approval (No. 1/2026, 1 August 2026) authorized the retrospective secondary analysis of pseudonymized clinical data from the center’s database and waived individual consent; the framework approval (No. 2/2026, 7 September 2026), as amended by Addendum No. 2, covers all retrospective secondary analyses of this database, including the present study. The waiver applies to secondary research analysis and dissemination, not to retrospective approval of the original clinical care.

## 3. Results

### 3.1. Population

The sequential flow was 8985 → 6598 → 2551 → 2540 → 920 → 800 → 760 (Table 1; Figure S1). The largest losses were the absence of an eligible T2/T3 examination and the T2 ≥ 22-week reference-domain restriction. Compared with the 8225 parent pregnancies not included in the primary cohort, the 760 included pregnancies were similar in maternal age, BMI, parity, smoking, T1 uterine-artery PI MoM and PAPP-A MoM; the largest standardized difference among these characteristics was for assisted reproduction (3.03% vs. 6.19%; SMD −0.151) (Table 2). Third-trimester follow-up itself was not universal: 2743/8985 (30.5%) parent pregnancies had a selected T3 examination before cleaning. However, among 2730 clean T3 examinations at 28–40 weeks, UA-PI was positive in 2659 (97.4%), MCA-PI in 2633 (96.4%) and both for CPR in 2592 (94.9%). CPR availability was 97.9% in T3-SGA, 95.0% in T3-AGA and 92.3% in T3-LGA fetuses. Thus, Doppler acquisition was near-routine once T3 imaging occurred, whereas selection into T3 follow-up remains a potential source of bias.

The internal CPR reference comprised 2592 selected examinations from 2592 pregnancies and 2408 women; 181 women contributed more than one pregnancy. The cEFW derivation reproduced the archived values exactly. In the 920-pregnancy derivation cohort, the joint linear/quadratic test for T3 gestational age was *p* = 0.909 for mean cEFW; after restriction to the 760 AGA pregnancies it was *p* = 0.0258. Re-estimating cEFW with linear and quadratic T3 gestational-age terms changed the primary width ratios minimally: 1.194 (95% CI 0.766–1.424) at CPR z −2, 1.124 (0.864–1.257) at z −1.5 and 0.970 (0.922–1.056) at z 0.

### 3.2. Third-Trimester CPR and Preceding Growth

The unconditional P10–P90 range of cEFW was −1.027 to +1.019 SD (width 2.046 SD). The conditional median increased by 0.417 SD (95% CI −0.211 to 0.694) from CPR z −2 to +1; this interval is wide because the estimate at z −2 rests on few pregnancies, whereas the linear mean association reported below was estimated more precisely. Point estimates of the conditional central 80% width showed little narrowing across the evaluated CPR range: width ratios ranged from 0.968 to 1.194, with 1.120 (95% CI 0.859–1.253) at CPR z −1.5 and 0.968 (0.926–1.058) at z 0 (Table 3, Figure 1 and Figure 2). At the low CPR tail, precision was limited: only seven pregnancies lay within ±0.25 SD of CPR z −2 and 44 within ±0.25 SD of z −1.5. Accordingly, the confidence intervals at low CPR allow both meaningful narrowing and widening and do not establish equivalence. Conditional AC velocity showed the same broad pattern, with width ratios of 1.103 (0.879–1.304) at z −1.5 and 1.042 (0.938–1.076) at z 0.

cEFW increased by 0.100 SD per 1 SD of CPR z (95% CI 0.040–0.160; *p* = 0.0011), and CPR explained 1.43% of cEFW variance. For the descriptive threshold cEFW ≤ −1 SD, there were 80 events and the AUC was 0.553 (95% CI 0.479–0.619). For cEFW ≤ −1.5 SD, there were only 23 events and the AUC was 0.635 (0.493–0.742); this interval is too wide to exclude moderate discrimination. These AUCs classify lower-tail measured conditional growth and should not be interpreted as prediction of placental insufficiency or adverse perinatal outcome.

Modeled probabilities of cEFW ≤ −1 SD decreased from 14.4% (95% CI 8.5–22.1) at CPR z −2 to 10.5% (8.4–12.9) at z 0 and 9.0% (6.1–12.3) at z +1, against an overall frequency of 10.5% (80/760). For cEFW ≤ −1.5 SD, the corresponding probabilities were 7.6% (2.3–15.1), 2.8% (1.4–4.0) and 1.7% (0.6–3.1), against 3.0% (23/760) overall (Table 4). Low CPR was therefore associated with a modest relative increase in the probability of lower-tail measured growth, but most fetuses with low CPR did not have lower-tail growth, and the probabilities at low CPR were imprecise. Throughout, “limited individual-level information” refers to these measured quantities: the central 80% width, discrimination and modeled lower-tail probabilities.

### 3.3. Sensitivity Analyses and Measurement Error

Sensitivity analyses supported the stability of the point estimates but emphasized uncertainty in the lower CPR tail. Across alternative 3-, 4- and 5-knot specifications, the width-ratio point estimate ranged from 1.148 to 1.195 at CPR z −2, from 1.102 to 1.123 at z −1.5 and from 0.968 to 1.007 at z 0; bootstrap intervals remained wide at low CPR. In the T3 ≥ 34-week subset (*n* = 151; median 34.57 weeks), width ratios were 1.428 (95% CI 0.691–2.027; local *n* = 3) at z −2, 1.239 (0.762–1.595; local *n* = 8) at z −1.5 and 0.977 (0.774–1.160) at z 0. At ≥36 weeks, only 22 pregnancies were available, with no CPR z ≤ −1.5 and no cEFW ≤ −1 SD events; spline or ROC estimates were therefore not considered clinically interpretable. Using the external Zohav reference, interpolated CPR MoM at the empirical 2.5th percentile (0.648) gave a width ratio of 1.062 (95% CI 0.811–1.276), and the median MoM (0.992) gave 0.975 (0.928–1.062); completed-week scaling gave similar results. Only 10–11/760 pregnancies fell below the published external p10 threshold, again limiting precision at the clinically low tail. Raw CPR in the 760 AGA pregnancies had a median of 2.08 (IQR 1.76–2.43), a 2.5th percentile of 1.39, a 5th percentile of 1.44 and a 10th percentile of 1.53; no value was ≤1.0. The published weekly medians at 32–34 weeks (2.03–2.20) were close to ours, but the published 10th percentiles (1.12–1.30; 0.55–0.62 MoM) lay far below them, reflecting wide dispersion in small weekly samples (27–40 examinations), whereas 92% of our T3 examinations were performed at 31–34 weeks. The numbers of pregnancies below the external 5th percentile were 5/760 (completed week) and 6/760 (interpolated). Internal CPR z −2 and −1.5 corresponded to raw CPR values of approximately 1.22–1.24 and 1.39–1.41 at 31–34 weeks; z −2 lay between the external 5th and 10th percentiles at 32 weeks, below both at 33 weeks and above the 10th percentile at 34 weeks. Using completed-week thresholds, 6 of the 8 pregnancies with internal z ≤ −2 were below the external 10th and 2 below the external 5th percentile, and all 11 below the external 10th percentile had internal z < −1.28. Differences in population, AGA restriction, Doppler technique, gestational-age distribution and reference-sample size could not be separated with these data.

Without AGA restriction (878 pregnancies from 857 women, including 51 SGA and 67 LGA fetuses at T3), the unconditional P10–P90 range of cEFW was wider (2.455 versus 2.046 SD) and the association was stronger: 0.248 SD per 1 SD of CPR z (95% CI 0.169–0.326), R^2^ 6.45%, AUC 0.615 (0.553–0.675) for cEFW ≤ −1 SD (125 events) and 0.691 (0.601–0.775) for cEFW ≤ −1.5 SD (56 events). The conditional median increased by 0.916 SD (0.616–1.216) from CPR z −2 to +1. Point-estimate width ratios nevertheless showed no narrowing at low CPR: 1.318 (1.002–1.525) at z −2, 1.183 (0.987–1.299) at z −1.5, 1.053 (0.929–1.128) at z −1, 0.950 (0.892–1.020) at z 0 and 0.979 (0.878–1.063) at z +1 (Table 4). Restriction to AGA fetuses therefore attenuated the mean association and discrimination, as expected when selection depends on T3 size, which also enters cEFW; however, point-estimate width ratios showed no narrowing at low CPR in either population.

In the central measurement-error scenario (4.3% relative EFW error CV), simulated error-only cEFW variance was equivalent to 74.8% of observed cEFW variance when errors were independent between examinations and 48.9% when their correlation was 0.5; when the observed conditional model was held fixed, the corresponding figures were 44.2% and 22.8%. Under the additional Gaussian signal/noise approximation, the width ratios attainable by an ideal predictor of error-free growth were 0.865 and 0.699, respectively (Figure 2B). Results varied materially across the 19 scenarios (Table S3).

## 4. Discussion

### 4.1. Principal Findings

In AGA fetuses, third-trimester CPR and preceding growth measured by serial ultrasound were associated at the population level, but the point estimates showed little narrowing of the central 80% of measured growth histories across the central and better-represented CPR range. At low CPR, where the clinically most abnormal values lie, confidence intervals were wide and clinically meaningful narrowing cannot be excluded. The same distinction applied to the ROC analysis: discrimination for cEFW ≤ −1 SD was weak, whereas the estimate for the more extreme ≤−1.5 SD threshold was too imprecise to exclude moderate discrimination. Modeled probabilities of lower-tail growth differed only modestly across CPR values. Without AGA restriction, the mean association and discrimination were stronger, but point-estimate width ratios likewise showed no narrowing at low CPR.

### 4.2. Association Is Not Individual Information

The clinical context is important. CPR is primarily a contemporaneous hemodynamic and prognostic marker, not a guideline-endorsed method for reconstructing previous fetal growth. Our question arose because CPR has also been described as a marker of impaired growth velocity or failure to reach growth potential [11,12,13,14]. We therefore tested the individual-level informational implication of that interpretation rather than proposing CPR as a substitute for serial scanning. The POP study showed that growth velocity can add clinically relevant information to fetal size in identifying small fetuses at increased neonatal risk [16], while MacDonald, Kennedy and others linked reduced velocity in normally sized fetuses with placental insufficiency or adverse characteristics [12,13,14]. Those studies ask whether growth history identifies biological or outcome risk; our study asks the inverse statistical question of how much a single CPR value constrains a preceding measured history.

The prognostic literature also shows why these questions should not be conflated. Meta-analyses have associated abnormal CPR with adverse perinatal outcomes, but predictive accuracy varies by outcome, population and threshold [5,6,7,10]. In an individual-participant-data meta-analysis of 18,731 participants, CPR alone and UA-PI alone had similar AUCs for a composite adverse outcome (0.778 and 0.775), and adding CPR to UA-PI increased the AUC by only 0.003 [9]. Conversely, other meta-analyses have found stronger associations for selected outcomes or suspected FGR populations [6,7]. None of these results implies that CPR should reconstruct a previous biometric trajectory, and our findings do not challenge the use of CPR for the separate purpose of contemporaneous risk assessment.

### 4.3. What Measurement-Error Changes

Each measured growth history contains the error of two scans, so even a perfect marker of true biological growth could not fully narrow the range of measured histories. Under the assumptions of our central simulation, the approximate width ratio attainable by an ideal predictor was 0.70–0.87, depending on temporal correlation of measurement error. This is an assumption-dependent benchmark, not a cohort-specific lower bound. HC contributes to the EFW formula and therefore its measurement reproducibility also matters. Mlodawski et al. reported good-to-excellent repeatability and reproducibility for HC and other skull measurements using a SonoCNS three-dimensional/AI-assisted acquisition workflow [30]. That study supports the general relevance of head-biometry reproducibility, but its acquisition method differs from routine two-dimensional measurements in our historical cohort and should not be used as a direct estimate of our HC error. CPR itself also has limited reproducibility [23], which could further attenuate observed associations under classical measurement-error assumptions but was not simulated here.

### 4.4. Clinical Interpretation

Two practical implications follow. First, where serial biometry is available, CPR and growth trajectory should be interpreted as complementary measurements: one describes current hemodynamics and the other measured change over time. Second, our data do not justify using a reassuring CPR to infer a stable previous trajectory, nor do they justify using a low CPR to infer that deceleration occurred. At very low CPR, however, the present data are too sparse to exclude useful information. The gestational timing further limits generalization: the median T3 examination was 32.86 weeks, so this study mainly captures growth between approximately 22 and 33 weeks rather than the 36–40-week interval in which late FGR may become clinically apparent. The ≥34-week sensitivity analysis was imprecise and the ≥36-week subgroup was too small for meaningful inference.

### 4.5. Strengths and Limitations

The strengths include a written analysis plan fixed before the primary analyses, conditional growth derived before AGA restriction, separation of mean association from distributional overlap, bootstrap intervals that repeat all calibration steps, a complementary published conditional-AC method, explicit sensitivity to spline specification and T3 gestational age, an externally scaled CPR sensitivity analysis and a measurement-error simulation. The near-complete availability of UA and MCA Doppler once a T3 examination occurred also argues against MCA being recorded only in obviously growth-restricted fetuses.

Several limitations are important. This study is retrospective and single-center, and third-trimester follow-up was not universal. Although the T3 examination was routinely offered, the database does not encode the reason for attendance or non-attendance, or whether an additional clinical indication was present; selection into longitudinal follow-up may therefore still affect the observed CPR–growth relationship. Although UA, MCA and DV Doppler were systematically acquired once T3 imaging occurred, the export does not preserve waveform-level repeats, T2/T3 operator identity or which specific prior findings were actively considered during each T3 examination. Internal CPR standardization is a study scale, not a clinical centile, and published CPR charts differ; the external sensitivity was therefore used as a robustness check rather than a replacement clinical calibration [9,24,25,26,27]. AC and EFW are not independent. Restriction to AGA fetuses was based on T3 EFW, which also enters cEFW; this truncation attenuated the mean association and discrimination relative to all size classes, so the primary estimates apply to fetuses already classified as AGA, the group in which inferring growth history from CPR is most tempting clinically. The low CPR tail and the ≥34-week subgroup were sparse, precluding equivalence-type conclusions. Finally, perinatal outcome data were incomplete and non-uniformly ascertained: birthweight was available for 276/760 primary pregnancies, 5-min Apgar for only 20/760 and umbilical-artery pH for none. A selected-case outcome analysis would therefore risk substantial ascertainment bias and was not added.

### 4.6. Future Directions

Future work should combine fixed-window serial biometry with repeated Doppler later in gestation, including a sufficiently large 36–40-week sample to estimate the low-CPR tail precisely. Repeated CPR and biometry in the same pregnancies would allow direct estimation of measurement repeatability rather than reliance on literature-based error assumptions. Prospective studies can then examine whether growth trajectory, fetal size, placental vascular indices, angiogenic markers and maternal hemodynamics provide complementary information about current fetal state and perinatal outcome [31,32], without attributing unexplained variance in the present study to any specific mechanism. Such integration of fetal growth assessment, placental vascular indices and maternal hemodynamics belongs within a broader multidisciplinary approach to pregnancy care that brings together biological, technological and organizational factors [33].

## 5. Conclusions

In this cohort of AGA fetuses examined mainly at 31–34 weeks, third-trimester CPR and preceding growth measured by serial ultrasound were associated. Across the central and better-represented CPR range, point estimates showed limited narrowing of the central 80% of measured growth histories; at very low CPR, clinically meaningful narrowing cannot be excluded because estimates were imprecise. Lower-tail growth probabilities differed only modestly across CPR, and restriction to AGA fetuses attenuated the association relative to all size classes. Measurement error may contribute substantially to the observed overlap. CPR should not be assumed to reconstruct measured growth history, but these data do not assess or diminish its separate prognostic role for adverse perinatal outcome.

## Figures and Tables

**Figure 1:**
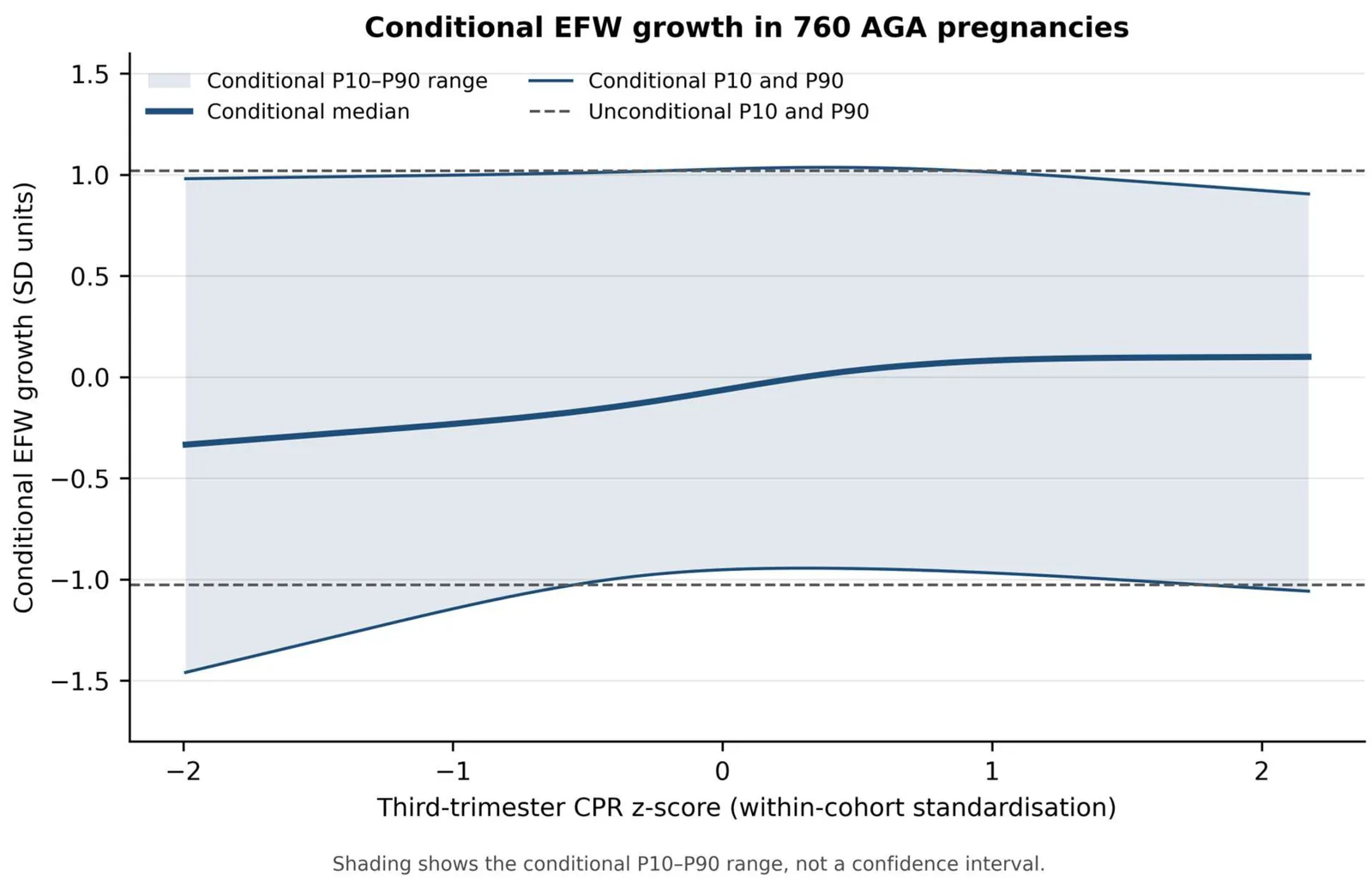
Conditional EFW growth against third-trimester CPR in 760 AGA fetuses. Solid blue lines show the fitted conditional 10th percentile, median (thicker line) and 90th percentile; shading shows the conditional 10th–90th percentile range, not a confidence interval. Dashed lines show the unconditional 10th and 90th percentiles. The fitted median shifts, while the point-estimate central 80% width changes little across the plotted CPR range; uncertainty in the width ratios is shown in Figure 2. Only aggregate model estimates are displayed. CPR is standardized for gestational age within the cohort. EFW, estimated fetal weight; CPR, cerebroplacental ratio; AGA, appropriate for gestational age; SD, standard deviation.

**Figure 2:**
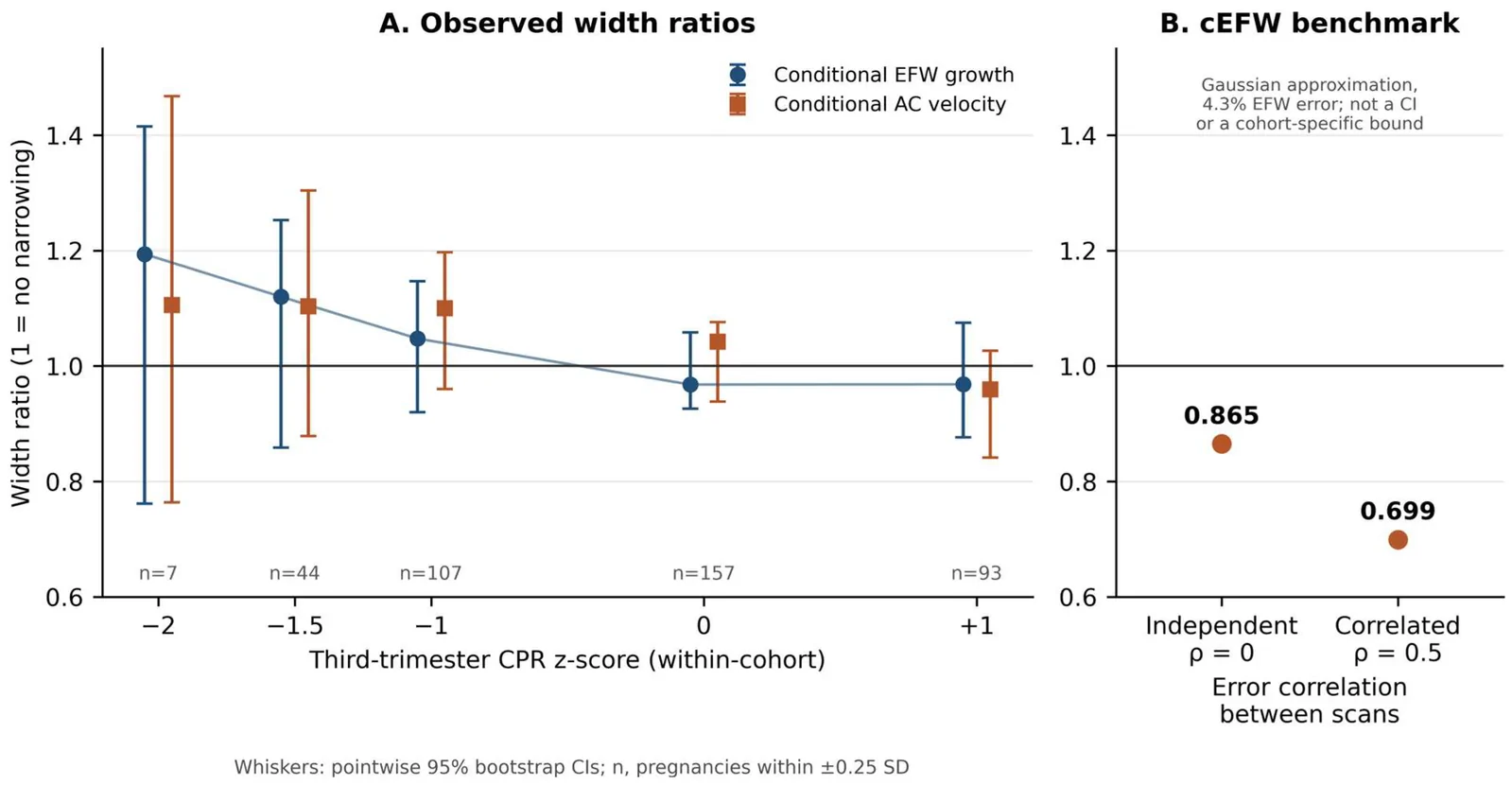
(**A**) Observed width ratios for conditional EFW growth and conditional AC velocity at five CPR z-scores. Whiskers are pointwise 95% bootstrap confidence intervals; the connecting line is a visual guide. Local *n* is the number of pregnancies within ±0.25 SD of each CPR position. (**B**) Gaussian approximation of the width ratio for an ideal predictor of error-free growth under a 4.3% relative EFW error CV, for between-examination error correlations ρ = 0 and 0.5. These cEFW-only benchmarks are neither confidence intervals nor bounds demonstrated in the cohort and do not apply to the AC or sensitivity analyses. AC, abdominal circumference; cEFW, conditional EFW growth; EFW, estimated fetal weight; CPR, cerebroplacental ratio; CV, coefficient of variation. The benchmarks use the re-estimated-model variance comparison in Supplementary Methods S1, not the fixed-model comparison.

**Table 1:** Sequential cohort selection from the T1-selected parent cohort to the primary AGA CPR analysis.

Step	*n*	Women	Excluded at Step/Reason
T1 selected parent	8985	8065	—; first eligible T1 record with positive UtA-PI MoM and PAPP-A MoM, fetus 1, no T1 multiple-pregnancy flag; 7 repeated eligible T1 examinations deduplicated
T2 biometry selected, 20 + 0–24 + 6	6598	5926	2387; 2360 without an examination in the window and 27 without positive HC and AC
T3 biometry selected, 28 + 0–41 + 6	2551	2366	4047; 4020 without an examination in the window and 27 without positive HC and AC
Finite T2/T3 EFW z and interval	2551	2366	0; no additional exclusions
Clean singleton/chronology	2540	2362	11; 2 later multiple-pregnancy flags and 9 duplicate maternal episodes on the same T1 date
T2 ≥ 22 + 0, EFW reference-valid	920	898	1620; selected T2 examination before 22 + 0 weeks
T3 ≤ 40 + 0, EFW reference-valid	920	898	0; no additional exclusions
T3 EFW AGA, p10–p90	800	784	120; T3 EFW outside p10–p90
Positive same-examination UA-PI and MCA-PI	760	745	40; missing or non-positive UA-PI and/or MCA-PI

EFW, estimated fetal weight; HC, head circumference; AC, abdominal circumference; AGA, appropriate for gestational age; UA, umbilical artery; MCA, middle cerebral artery; PI, pulsatility index. The 8985-parent cohort was already selected on first-trimester screening availability and singleton designation.

**Table 2:** Available baseline characteristics in primary included pregnancies versus the remaining T1-selected parent cohort.

Characteristic	Included (*n* = 760)	Excluded (*n* = 8225)	SMD
Maternal age, years, mean	31.724	31.575	0.031
BMI, kg/m^2^, mean	23.375	23.598	−0.050
Nulliparous, %	66.05	65.19	0.018
Current smoker, %	11.45	13.70	−0.068
Assisted reproduction, %	3.03	6.19	−0.151
T1 uterine-artery PI MoM, mean	1.073	1.086	−0.042
PAPP-A MoM, mean	1.147	1.150	−0.003

Values are available-case summaries; missing values were not imputed. SMD, standardized mean difference. Gestational ages at the selected T2/T3 visits are not compared here because those variables are part of the cohort-selection definition.

**Table 3:** Conditional estimated fetal weight (EFW) growth and abdominal circumference (AC) velocity across third-trimester cerebroplacental ratio (CPR) in 760 appropriate-for-gestational-age fetuses.

Outcome	CPR z	Local *n*	P10, P50, P90 (SD)	Width (SD)	Width Ratio (95% CI)
cEFW	−2.0	7	−1.46, −0.33, 0.98	2.443	1.194 (0.761–1.415)
cEFW	−1.5	44	−1.30, −0.28, 0.99	2.292	1.120 (0.859–1.253)
cEFW	−1.0	107	−1.14, −0.23, 1.00	2.143	1.047 (0.920–1.147)
cEFW	0.0	157	−0.95, −0.06, 1.03	1.980	0.968 (0.926–1.058)
cEFW	1.0	93	−0.97, 0.08, 1.01	1.981	0.968 (0.876–1.075)
AC	−2.0	7	−1.46, −0.06, 1.24	2.706	1.106 (0.764–1.467)
AC	−1.5	44	−1.38, −0.04, 1.32	2.700	1.103 (0.879–1.304)
AC	−1.0	107	−1.29, −0.01, 1.40	2.692	1.100 (0.960–1.197)
AC	0.0	157	−1.12, 0.18, 1.43	2.550	1.042 (0.938–1.076)
AC	1.0	93	−0.98, 0.33, 1.37	2.348	0.959 (0.841–1.026)

Local *n*: pregnancies within ±0.25 SD of the CPR value, not the sample size used to fit the model. Unconditional P10–P90: cEFW −1.027 to 1.019 SD (width 2.046); AC −1.061 to 1.386 SD (width 2.447). Width ratio = conditional/unconditional P10–P90 width and describes the central 80% only; it does not summarize the conditional median or distribution tails. CIs are pointwise percentile intervals from 2000 woman-level bootstrap replicates.

**Table 4:** Modeled probabilities of lower-tail conditional EFW growth in AGA fetuses and width ratios without AGA restriction, by third-trimester CPR z-score.

CPR z	P (cEFW ≤ −1 SD), AGA % (95% CI)	P (cEFW ≤ −1.5 SD), AGA % (95% CI)	Width Ratio, All Size Classes (95% CI)
−2.0	14.4 (8.5–22.1)	7.6 (2.3–15.1)	1.318 (1.002–1.525)
−1.5	13.3 (8.8–18.8)	6.0 (2.2–10.0)	1.183 (0.987–1.299)
−1.0	12.3 (9.0–16.1)	4.7 (2.1–6.8)	1.053 (0.929–1.128)
0.0	10.5 (8.4–12.9)	2.8 (1.4–4.0)	0.950 (0.892–1.020)
1.0	9.0 (6.1–12.3)	1.7 (0.6–3.1)	0.979 (0.878–1.063)

AGA, appropriate for gestational age (*n* = 760); all size classes, *n* = 878 (51 SGA, 67 LGA). Probabilities were modeled by logistic regression on CPR z (linear term); overall frequencies were 10.5% (80/760) for cEFW ≤ −1 SD and 3.0% (23/760) for ≤−1.5 SD. Pregnancies within ±0.25 SD of each CPR value in the all-size cohort: 9, 58, 117, 170 and 115. All CIs are pointwise percentile intervals from the same 2000 woman-level bootstrap replicates (including re-standardization of CPR and re-derivation of cEFW) used for Table 3.

## Data Availability

The pseudonymized dataset is held by the Maternal-Fetal Medicine Center, Timișoara, and is not publicly available because of patient-confidentiality restrictions; aggregate data supporting the findings are available from the corresponding author on reasonable request. Analysis scripts and aggregate outputs are provided in Supplementary Code; no individual-level clinical records are included.

## Supplementary Materials

The following supporting information can be downloaded at: https://www.mdpi.com/article/10.3390/diagnostics16213442/s1, Figure S1: Cohort flow; Figure S2: Aggregate conditional growth categories in the 10th–15th CPR band; Table S1: Cohort characteristics; Table S2: Sensitivity analyses; Table S3: Measurement-error scenarios; Table S4: Exploratory analysis of second-trimester uterine artery pulsatility index and maternal characteristics; Table S5: Conditional growth categories within descriptive CPR bands. Supplementary Methods S1 describes the measurement-error simulation. Supplementary Code contains analysis scripts and aggregate outputs, without individual-level records.
