# Supplementary material for: Third-Trimester Cerebroplacental Ratio and Preceding Fetal Growth in Appropriate-for-Gestational-Age Fetuses: Limited Individual-Level Information in a Longitudinal Cohort
Source: Diagnostics (Basel). 2026 Oct 22;16(21):3442. doi: 10.3390/diagnostics16213442 (PMC13761876; doi:10.3390/diagnostics16213442)
Supplement: Supplementary file 1 [file diagnostics-16-03442-s001.zip › Supplementary_CPR-Histories_R2_Diagnostics.pdf]

Supplementary Materials

Third-Trimester Cerebroplacental Ratio and Preceding Fetal Growth in Appropriate-for-Gestational-Age Fetuses: Limited Individual-Level Information in a Longitudinal Cohort

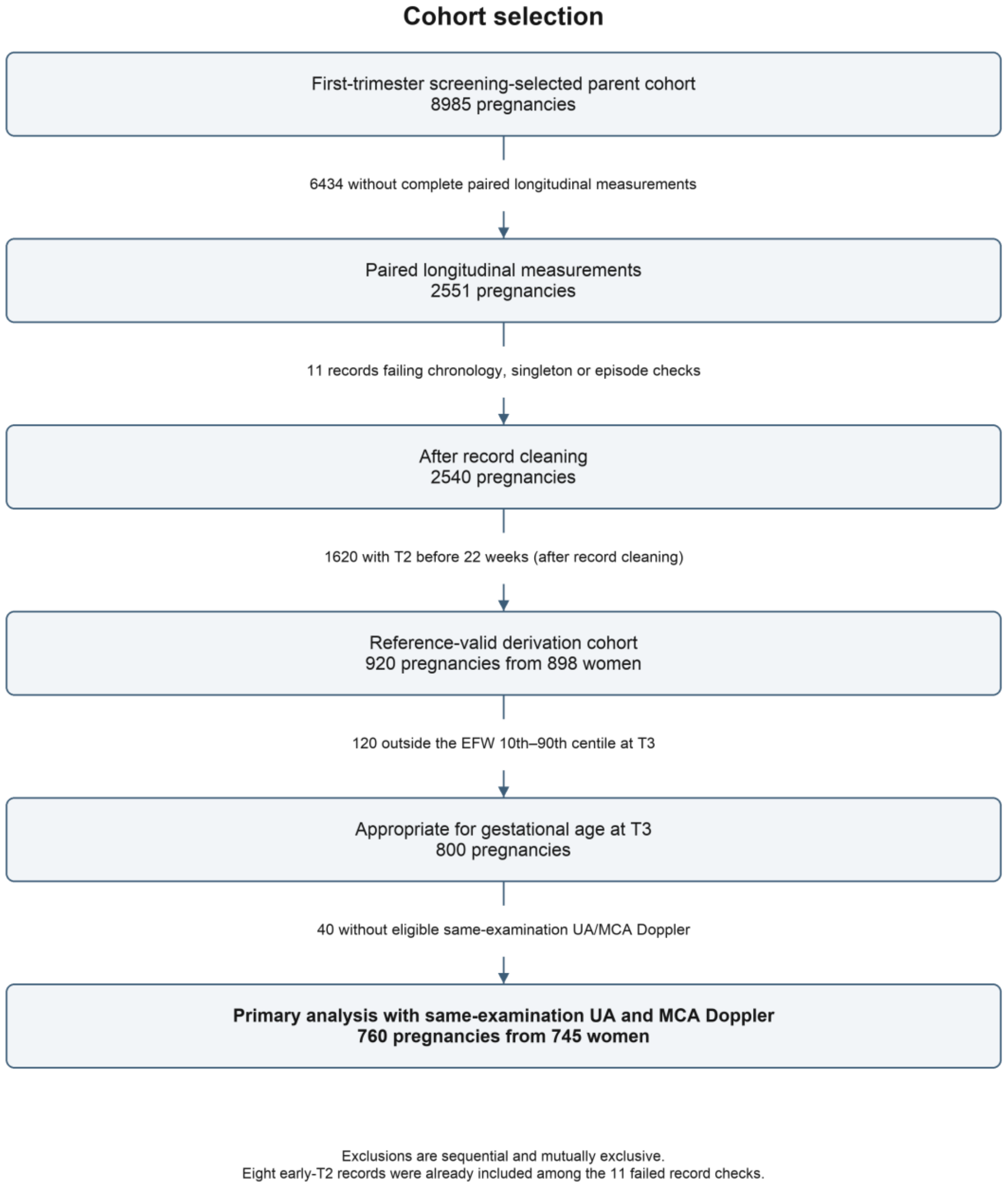

Figure S1. Sequential cohort selection. Counts of exclusions are mutually exclusive: eight pregnancies with T2 before 22 weeks were already removed at the record-cleaning step. Of 920 derivation pregnancies, 800 were AGA at T3 and 760 had eligible same-examination UA and MCA Doppler. AGA, appropriate for gestational age; EFW, estimated fetal weight; T2/T3, second/third trimester; UA/MCA, umbilical/middle cerebral artery.

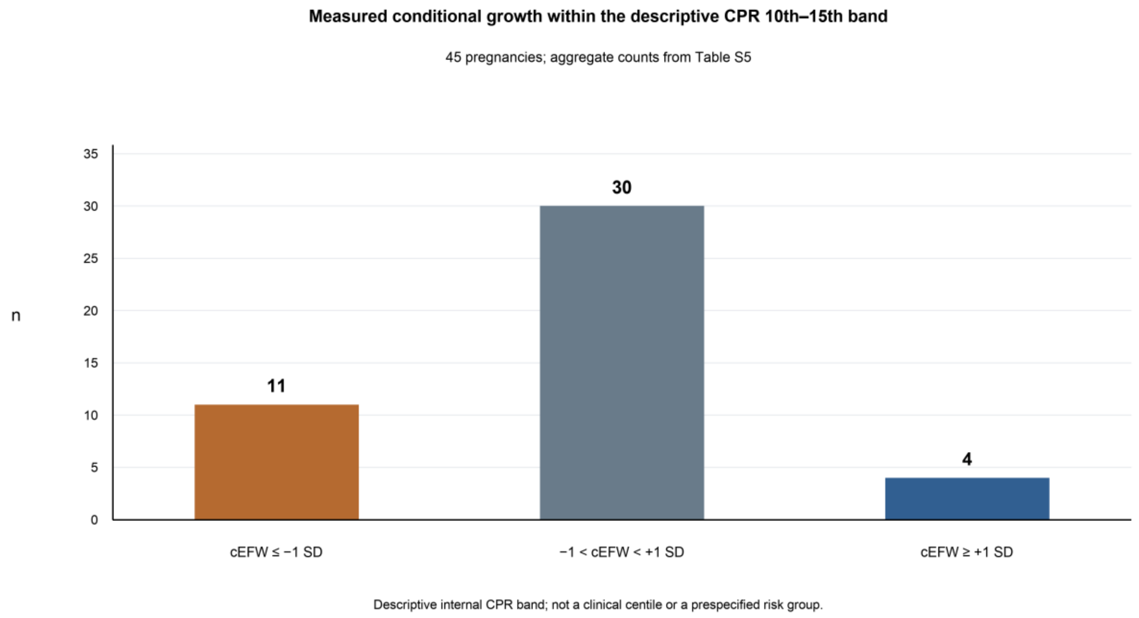

Figure S2. Aggregate conditional EFW growth categories among the 45 fetuses in the descriptive 10th–15th CPR band: cEFW ≤ −1 SD, 11; −1 < cEFW < +1 SD, 30; cEFW ≥ +1 SD, 4. Counts reproduce Table S5. The band is defined using standard-normal quantiles of the within-cohort CPR z-score, not empirical centiles of the AGA subset or externally validated clinical centiles. It was examined in earlier descriptive work and was not used for inference. The growth categories describe ultrasound measurements, which include measurement error. EFW, estimated fetal weight; cEFW, conditional EFW growth; CPR, cerebroplacental ratio; AGA, appropriate for gestational age; SD, standard deviation.

Table S1. Cohort characteristics. Values are median (interquartile range) unless stated.

Characteristic	Derivation cohort (n = 920; 898 women)	Analytic AGA cohort (n = 760; 745 women)
Gestational age at T2, weeks	22.43 (22.14–22.86)	22.43 (22.14–22.86)
Gestational age at T3, weeks	32.86 (32.29–33.71)	32.86 (32.29–33.71)
T2–T3 interval, weeks	10.29 (9.71–11.14)	10.29 (9.71–11.00)
EFW z-score at T2	0.03 (-0.61–0.58)	0.03 (-0.59–0.54)
EFW z-score at T3	-0.03 (-0.52–0.59)	-0.04 (-0.47–0.48)
CPR (MCA-PI/UA-PI)	2.07 (1.74–2.45)	2.08 (1.76–2.43)
Maternal age, years	31.34 (28.58–34.84)	31.32 (28.55–34.68)
Body mass index, kg/m²	22.49 (20.44–25.34)	22.50 (20.42–25.24)
Nulliparous, n (%)	602 (65.4%)	502 (66.1%)
Current smoker, n (%)	107 (11.6%)	87 (11.4%)
AGA at T3, n	800	760

CPR was available in 878/920 derivation pregnancies and all 760 analytic pregnancies; BMI was available in 915/920 and 755/760, respectively. Other characteristics in this table were complete. AGA refers to EFW at the third-trimester examination, not birthweight. EFW, estimated fetal weight; CPR, cerebroplacental ratio; BMI, body mass index.

Table S2. Sensitivity analyses at CPR z −1.5 and 0. Values are point estimates except where a pointwise 95% CI is specified.

Analysis	n	CPR z −1.5	CPR z 0
Primary cEFW	760	1.120 (0.859–1.253)	0.968 (0.926–1.058)
Conditional AC velocity	760	1.103 (0.879–1.304)	1.042 (0.938–1.076)
All fetal-size classes	878	1.183 (0.987–1.299)	0.950 (0.892–1.020)
One pregnancy per woman	745	1.066	0.989
Narrow interval (9.71–11.14 weeks)	430	0.934	1.013
Global residual SD	760	1.112	0.977
Historical window, cEFW	2119	0.958	1.003
Historical window, conditional AC	2119	0.959	1.017
Epoch 2010–2014	153	0.930	1.035
Epoch 2015–2019	165	0.743	0.998
Epoch 2020–2022	202	1.231	0.949
Epoch 2023–2026	240	1.157	0.960

The narrow interval is the interquartile range of the derivation cohort. Historical-window cEFW uses EFW reference extrapolation below 22 weeks and is a sensitivity analysis only. CPR calibration and growth scores were derived as described in the main Methods; epoch-specific quantile profiles were fitted within each epoch. Local counts within ±0.25 SD at CPR z −1.5 were 7, 10, 15 and 12 across successive epochs; at z 0 they were 31, 37, 46 and 43. At CPR z −2, the corresponding counts were 1, 1, 0 and 5, underscoring the sparse low-CPR support. Apart from the primary cEFW, conditional AC and all-size rows, no confidence interval is implied by these point estimates; all-size intervals come from the same 2000 woman-level bootstrap replicates as the primary analysis (main Table 4).

Supplementary Methods S1

The measurement-error benchmark preserves gestational-age pairs, examination intervals and Doppler availability from the 920 derivation pregnancies. Each simulated fetus tracks the same latent EFW z-score at both examinations. The observed baseline z-score distribution is used as a working latent distribution; it is not assumed to be an error-free estimate. Two sensitivity scenarios multiply its dispersion by 0.75 or 1.25.

Random errors are lognormal multipliers with mean 1, parameterised by the stated relative coefficient of variation (CV). Correlations of 0 or 0.5 are imposed on the log-error scale between examinations. The central direct-EFW CVs are 2%, 4.3% and 6%; 0.4%, 10% and 21% are stress scenarios. The 4.3% value is informed by Dudley et al. [13 in the main manuscript]; the chosen CV range is not a published confidence interval. Transfer from that study's Hadlock EFW setting to this cohort's INTERGROWTH EFW and earlier examinations is an assumption.

For biometric-error scenarios, the AC/head-circumference (HC) limits of agreement from Sarris et al. [14 in the main manuscript] are converted to single-measurement SD using $LOA/(1.96\sqrt{2})$, assuming independent replicate errors of equal variance. Intraobserver CVs are 1.91% for AC and 1.08% for HC; interobserver CVs are 3.17% and 1.77%. Between-biometry log-error correlation is 0 or 0.5. Direct EFW error is not added on top of AC/HC error in the same scenario.

The EFW mean model and gamma/log residual-variance model are re-estimated in each simulation before AGA selection. AGA is selected on the error-perturbed T3 EFW, then Doppler availability is applied; the resulting analytic n is not constrained to 760. For the variance comparison, simulated residuals are expressed in the conditional-SD scale of the observed data rather than normalised by their own simulated SD.

Let f be simulated residual variance on the observed scale divided by observed analytic cEFW variance (0.605383). This is a comparison between simulated and observed settings, not an identified decomposition of real variance. Under an additional independent additive signal–noise model with Gaussian, homoscedastic components, the ideal-predictor P10–P90 width ratio is approximately $\sqrt{f}$. It is not a universal or CPR-position-specific lower bound. Scenarios with $f \geq 1$ exceed the observed variance budget; no valid ideal-predictor width ratio is reported for them.

An alternative comparison holds the observed mean model fixed and compares error-perturbed with error-free scores; it estimates a different quantity and is reported alongside the re-estimated-model comparison. Nineteen scenarios were run with 500 repetitions each. Scenario entries are means across repetitions. Monte Carlo variation does not quantify uncertainty about the true error magnitude or biology. The analysis does not simulate CPR error.

Table S3. All 19 measurement-error scenarios (500 repetitions each).

Error scenario	ρ time	ρ AC–HC	Variance ratio re-estimated (%)	Variance ratio fixed (%)	Approximate WR
EFW CV 0.4%	0.0	—	1.0	0.4	0.100
EFW CV 2%	0.0	—	22.6	9.8	0.476
EFW CV 2%	0.5	—	13.6	5.0	0.368
EFW CV 4.3%	0.0	—	74.8	44.2	0.865
EFW CV 4.3%	0.5	—	48.9	22.8	0.699
EFW CV 6%	0.0	—	106.8	83.6	Not estimable
EFW CV 6%	0.5	—	76.0	43.5	0.872
EFW CV 10%	0.0	—	144.4	212.2	Not estimable
EFW CV 21%	0.0	—	158.4	746.1	Not estimable
AC/HC intraobserver	0.0	0.0	52.9	36.1	0.727
AC/HC intraobserver	0.0	0.5	62.4	43.6	0.790
AC/HC intraobserver	0.5	0.0	29.2	22.1	0.540
AC/HC intraobserver	0.5	0.5	35.1	26.7	0.593
AC/HC interobserver	0.0	0.0	107.9	90.5	Not estimable
AC/HC interobserver	0.0	0.5	120.3	107.3	Not estimable
AC/HC interobserver	0.5	0.0	68.9	56.0	0.830
AC/HC interobserver	0.5	0.5	80.6	66.2	0.898
EFW CV 4.3%; latent ×0.75	0.0	—	66.3	43.7	0.814
EFW CV 4.3%; latent ×1.25	0.0	—	83.3	44.5	0.913

Variance ratios are simulated/observed comparisons, not identified percentages of real measurement error. “Fixed” refers to the alternative observed-model comparison. WR, width ratio; CV, coefficient of variation; ρ time, correlation between examination errors; ρ AC–HC, correlation between biometric errors. “Not estimable” indicates $f \geq 1$. Approximate WR applies only to cEFW under the assumptions in Supplementary Methods S1. The 0.4%, 10% and 21% EFW scenarios and the altered latent dispersions are stress tests.

Table S4. Exploratory analysis (specified after the primary results): variance in third-trimester CPR z explained by conditional growth, second-trimester uterine artery PI and maternal characteristics. Nested ordinary least-squares models fitted on identical pregnancies within each set.

Set / model	Pregnancies (women)	R^2, % (95% CI)	Added R^2, percentage points (95% CI)
A: cEFW	391 (388)	0.449 (0.002–2.765)	—
A: + uterine artery PI (log MoM)	391 (388)	0.602 (0.051–3.640)	0.153 (0.0004–2.072)
B: cEFW	388 (385)	0.309 (0.001–2.449)	—
B: + uterine artery PI	388 (385)	0.594 (0.044–3.540)	0.284 (0.0008–2.373)
B: + age, BMI, nulliparity, smoking	388 (385)	1.445 (0.835–6.059)	0.851 (0.286–4.261)
Post hoc: cEFW + gestational-age-adjusted log raw uterine artery PI	720	1.33 → 1.42	0.08

Uterine artery PI MoM was recorded in 391 of 760 pregnancies and raw PI in 720. R^2 is apparent in-sample R^2; because R^2 cannot decrease when predictors are added, intervals for added R^2 are descriptive and cannot include negative values. T3 UA-PI and MCA-PI were not used as predictors.

Sets A and B use complete cases for their respective predictor blocks. Uterine-artery PI MoM was log-transformed. Maternal covariates were age, BMI, nulliparity and current smoking. For the additional raw-PI analysis, log PI was adjusted internally for T2 gestational age. Results are exploratory; a predictor block's apparent in-sample ΔR^2 is not a test of biological contribution. MoM, multiple of the median; PI, pulsatility index; BMI, body mass index.

Table S5. Conditional growth categories in five selected descriptive CPR bands.

CPR band (within-cohort normal score)	n	cEFW ≤ −1 SD	−1 < cEFW < +1 SD	cEFW ≥ +1 SD
<5th	14	1 (7.1%)	12 (85.7%)	1 (7.1%)
5th–10th	38	3 (7.9%)	30 (78.9%)	5 (13.2%)
10th–15th	45	11 (24.4%)	30 (66.7%)	4 (8.9%)
15th–20th	47	6 (12.8%)	39 (83.0%)	2 (4.3%)
40th–60th	161	18 (11.2%)	128 (79.5%)	15 (9.3%)

The five selected bands comprise 305 pregnancies and do not partition the full analytic cohort of 760. Boundaries are obtained from standard-normal quantiles of the internally standardised CPR z-score; they are not empirical centiles of the AGA subset or externally validated clinical centiles. Percentages use each row's n. These summaries were not used for inference. The 10th–15th band had been selected in earlier descriptive work, so its elevated observed proportion must not be treated as evidence of a distinct risk category or as a prespecified comparison.
